# Evaluation of Influence of Various Polymers on Dissolution and Phase Behavior of Carbamazepine-Succinic Acid Cocrystal in Matrix Tablets

**DOI:** 10.1155/2015/870656

**Published:** 2015-08-24

**Authors:** Majeed Ullah, Hanif Ullah, Ghulam Murtaza, Qaisar Mahmood, Izhar Hussain

**Affiliations:** ^1^Department of Pharmacy, COMSATS Institute of Information Technology, Abbottabad 22060, Pakistan; ^2^Department of Environmental Sciences, COMSATS Institute of Information Technology, Abbottabad 22060, Pakistan

## Abstract

The aim of current study was to explore the influence of three commonly used polymers, that is, cellulosics and noncellulosics, for example, Methocel K4M, Kollidon VA/64, and Soluplus, on the phase disproportionation and drug release profile of carbamazepine-succinic acid (CBZ-SUC) cocrystal at varying drug to polymer ratios (1 : 1 to 1 : 0.25) in matrix tablets. The polymorphic phase disproportionation during in-depth dissolution studies of CBZ-SUC cocrystals and its crystalline properties were scrutinized by X-ray powder diffractrometry and Raman spectroscopy. The percent drug release from HPMC formulations (CSH) showed inverse relation with the concentration of polymer; that is, drug release increased with decrease in polymer concentration. On contrary, direct relation was observed between percent drug release and polymer concentrations of Kollidon VA 64/Soluplus (CSK, CSS). At similar polymer concentration, drug release from pure carbamazepine was slightly lower with HPMC formulations than that of cocrystal; however, opposite trend in release rate was observed with Kollidon VA/64 and Soluplus. The significant increase in dissolution rate of cocrystal occurred with Kollidon VA/64 and Soluplus at higher polymer concentration. Moreover, no phase change took place in Methocel and Kollidon formulations. No tablet residue was left for Soluplus formulation so the impact of polymer on cocrystal integrity cannot be predicted.

## 1. Introduction

Solubility and permeation features define the bioavailability of orally administered drugs [1]. Based on these attributes, biopharmaceutical classification system (BCS) categorizes drugs into four different classes, I–IV. Although BCS class II drugs exhibit poor solubility, they can escape easily into the gastrointestinal tract (GIT), while dissolution rate defines the rate and degree of absorption of class II drugs. As a result, the focal point of ongoing research is to improve the dissolution or release of these drugs from the formulations [2, 3]. Dissolution rate of a drug, as described by Noyes-Whitney equation, is mainly controlled by the surface area of the particles, bulk concentration of the drug, and the diffusion layer in addition to the width of the diffusion layer [4]. Therefore, better dissolution of a drug can be obtained by tuning any one of these parameters. For a drug's dissolution enhancement, most commonly utilized approach is modification of the drug's solid form or using suitable excipients in devising the suitable formulations [5, 6].

The use of solid forms with higher aqueous solubility has been an effective strategy for improving drug bioavailability. The salt formation of the API is widely adopted strategy for improving its solubility; but limitation of the process is its inapplicability to nonionizable drugs and also its stability concerns in solid state [7]. Instead, API amorphous form may be used, since the apparent solubility of crystalline form is not better than that of the amorphous form; however, the solid state stability concerns and problems related to scale-up methods still present barriers to formulation development [8]. Due to the reasons, current research focus in solid drug screening programs involves the cocrystal form. Cocrystals are like other solid crystalline forms, since the equilibrium solubility of the drug is more relatively enhanced than its apparent solubility, also with the additional advantage that cocrystals may be formed from nonionizable drugs [9–11]. Since proton transfer is not required in this approach, the chemical space that can be taped for preparing cocrystals is significantly larger [12], especially with the recent advancement in forming carboxylic acids based (CAB) and pseudo-CAB cocrystals [13, 14]. Reported advantages offered by cocrystallization include superior stability under stress condition [12], enhanced purification efficiency [15], improvement in chemical stability [16], superior solid state stability than salts [17], improved dissolution performance, enhanced bioavailability [18], and better tabletability [19, 20].

In current study, BCS class II drug, carbamazepine (CBZ), was chosen as a model drug. It has very low aqueous solubility, that is, 170 *μ*g/mL, at room temperature [21, 22]. Moreover, CBZ has four different known anhydrous polymorphic phases and dihydrate forms, and the mainly stable anhydrous form at ambient conditions is CBZ III polymorph aqueous solubility of CBZ form III is 380 *μ*g/mL, while that of dihydrate is around 130 *μ*g/mL at 25°C [23]. In aqueous solution, the CBZ polymorphs I–III transform to the stable dihydrate form [24]. In literature, the formation of CBZ cocrystals with quite a few coformers has been demonstrated [25–27]. A diprotic acid such as succinic acid (SUC) having pKa = 4.21 and 5.64 was the coformer chosen for this study.

Carbamazepine-succinic (CBZ-SUC) cocrystals are more soluble than the stable dihydrate form at pH 3 [28, 29]. Similar results were observed in our IDR studies and solution stability studies of this cocrystal system (data not shown here). Though pharmaceutical cocrystals can provide a superior dissolution profile and better apparent solubility to enhance the bioavailability of weakly water soluble drug, a key drawback of this approach is the recrystallization of drug's stable form during cocrystal's dissolution. It results in the failure of the superior drug properties [30]. The crystal growth rate and nucleation are greatly affected by excipients. That is why screening of stabilizing agent(s) is important in developing a formulation containing a highly soluble cocrystal of poorly water soluble APIs. In particular, the use of polymers to stabilize cocrystals against disproportionation during dissolution is a topic of enormous interest. As demonstrated in a number of studies, the stabilizing effects of polymers are mainly of kinetic nature, where specific API-polymer interactions in solution or the adsorption of the polymer on the nucleus or the growing crystal is possible mechanism [31–33]. Such effects of polymers are vital for sustaining supersaturation and avoiding genesis of poorly soluble API during dissolution.

Earlier studies have shown that hydroxypropyl methylcellulose (HPMC) inhibits the transformation of CBZ III to CBZ dihydrate in the gel film of tablets and in aqueous mediums [34]. The mechanism of this inhibitory effect of HPMC was due to hydrogen bonding between drug and polymer. Soluplus, a polymeric solubility enhancer, is a graft copolymer especially designed for preparation of solid solutions. The solid solution of Soluplus has significantly enhanced the solubility and bioavailability of several model drugs including CBZ (Ali et al, 2010) [35]. The oral bioavailability of water insoluble drugs such as nifedipine, tolbutamide, and indomethacin has been increased by formulating with water soluble polymer, Kollidon VA/64 (Forster et al., 2001). Thus, we selected HPMC, Kollidon VA/64, and Soluplus in our polymer screening studies. The influence of these polymers on phase conversions of the CBZ-SUC cocrystal and its crystalline properties were observed by X-ray powder diffraction (PXRD) and Raman spectroscopy.

When CBZ-SUC cocrystals are added to water, they transform to CBZ dihydrate (CBZ-DH), which is an indication of exceed in solution concentration of cocrystal than the solubility of CBZ-DH. However, the supersaturated solution of CBZ is metastable in nature, and CBZ can easily be crystallized from such a solution. The supersaturated state, that is, expected outcome of dissolution of CBZ-SUC cocrystals, must be maintained for a therapeutically relevant period of time in order for the drug to be absorbed. Thus, a drug crystallization inhibitor is required to maintain a supersaturated state [36]. Obviously, careful selection of the excipients is an essential part of successful product development. Thus, more research studies are needed to explore the functions of excipients on phase transition of cocrystals in order to maximize the benefits offered by the cocrystals. There is a dire need for using cocrystals as an alternative solid form in preformulation studies; however, the selection of a cocrystal strategy at the preclinical formulation stage in comparison to other formulation techniques for improving bioavailability is rarely adopted.

## 2. Materials and Methods

### 2.1. Materials

Soluplus (Lot number: 65511368E0) and polyvinylpyrrolidone vinyl acetate copolymer (PVP-VA), that is, Kollidon VA 64 (Lot number: 46581856Po), were procured from BASF SE, Germany. Methocel premium (CR, USA) was procured from Dow Chemicals, Michigan. Carbamazepine (Lot number: SLBB3655V) was purchased from Sigma Aldrich, USA. Magnesium stearate (Lot number: J03970) was procured form Mallinckrodt, USA.

### 2.2. Synthesis of Cocrystals

A solvent-drop grinding method (liquid assisted grinding (LAG)) was used for CBZ-SUC cocrystal preparation as a quick method during dissolution studies. A 2 : 1 stoichiometric mixture of CBZ (4.725 g) and NIC (1.181 g) was placed in mortar, crushed, and ground vigorously for 30 minutes with continuous dilutions with methanol [26].

### 2.3. Characterization of Cocrystals

**Figure 1:**
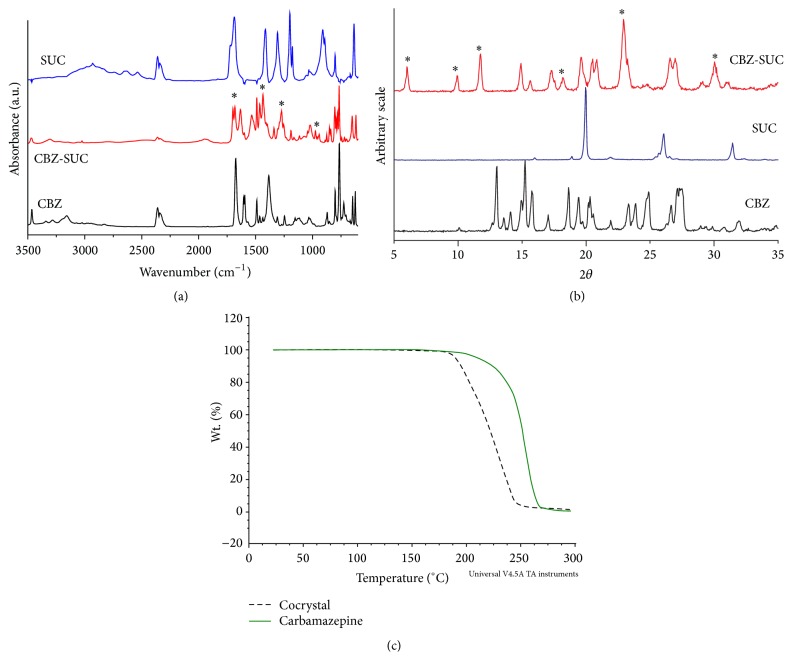
Solid state characterization of cocrystal. (a) FTIR of CBC, SUC, and CBZ-SUC. (b) XRD of CBZ, SUC, and CBZ-SUC. (c) TGA of CBZ and cocrystal.

Pure drug, coformer, and the cocrystals obtained from liquid assisted grinding were subjected to X-ray powder diffraction (PXRD), attenuated total reflectance Fourier transform infrared (AT-FTIR) spectroscopy, and thermogravimetric analysis (TGA). Characteristic peaks of cocrystals are denoted by “*∗*” in Figure 1.

#### 2.3.1. Powder X-Ray Diffraction

X-ray diffraction patterns of the samples were taken with Bruker-AXS D5005 diffractometer, with 2200 W closed copper source (1.54056 Å) calibrated with silicon standard [37]. Readings were recorded in the 2*θ* range between 5 and 35° having step size of 0.02°. The tube amperage and voltage were 40 mA and 40 kV, respectively, and dwell time was set at 2 s/step. The data are plotted by importing data to OriginLab (v.8; Northampton, MA, USA).

#### 2.3.2. IR Spectrometry

The IR spectra of the CBZ, SUC, and CBZ-SUC powder samples were collected utilizing a high resolution FTIR spectrometer (VERTEX 70, Bruker Optics Inc., Billerica, MA, USA). IR data were recorded by OPUS software (v5.5; Bruker Optics Inc., Billerica, MA, USA) and the data were further processed in OriginLab (v.8; Northampton, MA, USA).

#### 2.3.3. Thermogravimetric Analysis (TGA)

Powder sample's TGA data were collected by thermogravimetric analyzer (Model Q50 TGA, TA Instruments, New Castle, DE, USA). Around 3 mg of powder was heated in an open aluminum pan from room temperature to 300°C at 10°C/min under continuous dry nitrogen purge (75 mL/min). The data were analyzed by commercial software (Universal Analysis 2000, TA Instruments, New Castle, DE, USA).

### 2.4. Formulation of Matrix Tablets

Matrix tablet of both cocrystal and CBZ was prepared with HPMC, Soluplus, and Kollidon VA 64 at varying drug to polymer ratios as shown in Tables 1 and 2. All blends were compressed with Zwick, USA, using a 10 mm cylindrical tooling by applying 10 kN compression force after sieving; tablet tooling was lubricated with magnesium stearate before compression. All formulations had CBZ concentration of 200 mg per tablet.

### 2.5. Evaluation of Formulated Tablets

The weight variation, thickness, diameter, and hardness tests were performed for all formulations according to the compendial procedure. Hardness of tablets was checked by TA-XT2i texture analyzer (Texture Technologies Corporation, USA). As shown in table, all formulations were found to be in satisfactory acceptable limits as shown in Tables 3 and 4.

### 2.6. *In Vitro* Drug Release Study

*In vitro* drug release study was performed in 700 mL of 1% sodium lauryl sulphate (SLS) at 37°C as compendial medium; paddle speed was set at 75 rpm in USP apparatus type II. Sample of 5 mL was taken at predetermined intervals (1, 2, 3, 4, 5, and 6 hours). Then, CBZ concentration in solutions was measured using a UV-Vis spectrometer (DU 530 UV/vis spectrophotometer; Beckman Coulter, Chaska, MN, USA) at *λ*_max_ = 288 nm based on a separately constructed calibration curve (*R* ^2^ = 0.998, *y* = 0.44*x* + 0.05) [38].

The drug release data were fitted to various kinetics models, such as zero order [39], first order [40, 41], Higuchi's model [42], and Korsmeyer-Peppas model, also known as power law [43]. Consider

(1) Mt=Mo−Kot,ln⁡Mt=ln⁡Mo−K1t,Qt=KHt1/2,log⁡MtMf=log⁡ k+n log⁡ t,

where *M*_*t*_ is amount of undissolved drug at time (*t*), *M*_*o*_ is amount of undissolved drug at time (*t*) = 0, *t* is time of sampling, *K*_*o*_ is the zero-order release rate constant, *K*_1_ is the first-order release rate constant, *M*_*f*_ is amount of drug released at infinite time, *Q*_*t*_ is undissolved drug quantity at time (*t*), *K*_*H*_ is Higuchi's release rate constant, *k* is the release rate constant for power law, and *n* is release exponent for power law.

## 3. Results and Discussion

Phase purity of all powder samples was checked by X-ray powder diffractometry, IR spectrometry, and thermogravimetric analyzer. The PXRD confirmed that LAG transformed individual coformers to the cocrystal having typical diffraction peaks at 2*θ* = 5.8, 9.7, 11.5, 14.7, 22.8, and 29.9 [26]. Similarly, IR spectra also confirm shifting of peak in its spectra from CBZ to CBZ-SUC at regions 3000–3500 cm^−1^ and 1600–1700 cm^−1^ [29]. Moreover, TGA data also confirmed cocrystal formation when compared with published reference data (Figure 1). Different physical parameters of cocrystal and CBZ tablets were checked and found to be in acceptable range as shown in Tables 3 and 4.

**Figure 2:**
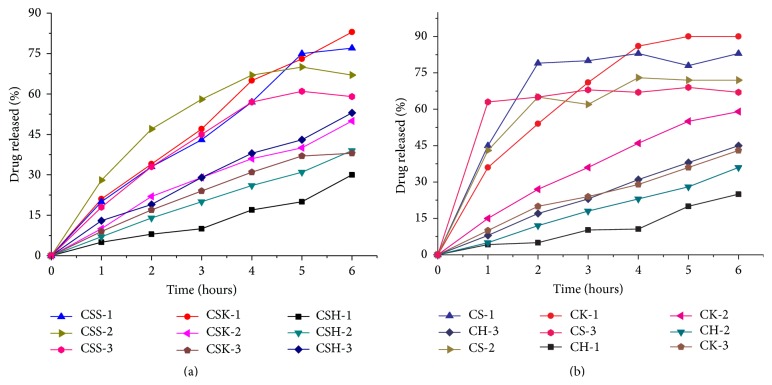
(a) Dissolution of cocrystal formulations with different polymers. (b) Dissolution of CBZ formulations with different polymers.

**Figure 3:**
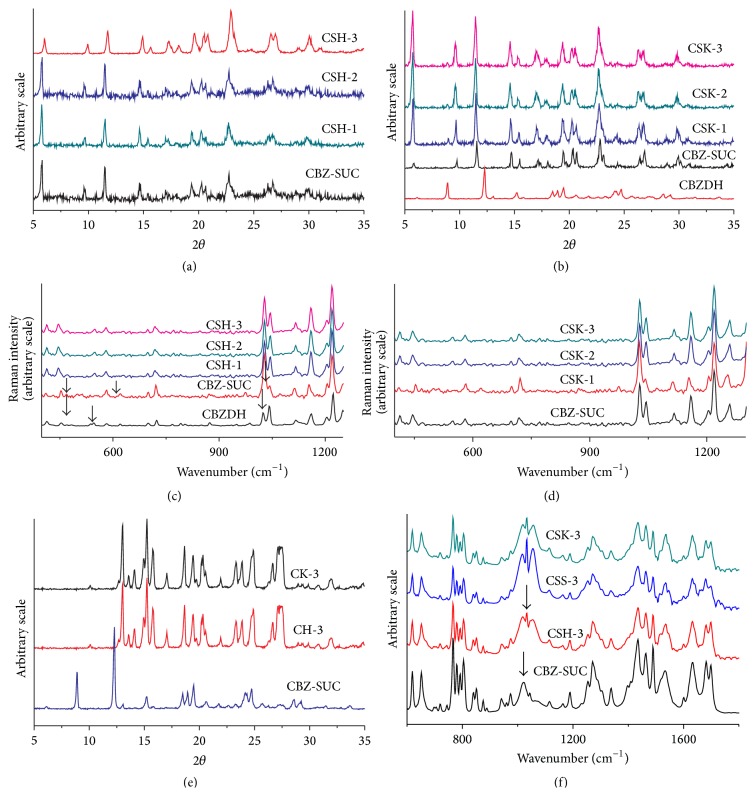
(a) XRD of CSH formulations after dissolution. (b) XRD of CSK formulations after dissolution. (c) Raman spectra of CSH formulations after dissolution. (d) Raman spectra of CSK formulations after dissolution. (e) XRD of CBZ after stability from CH-3/CK-3 formulations after dissolution studies. (f) FTIR of all cocrystal formulations at 1 : 4 concentration.

Interesting* in vitro* data were obtained when CBZ and cocrystals were compressed with polymers at different drug to polymer ratios (Tables 1 and 2). Initially 1 : 1 cocrystal/polymer ratio was selected to study the effect of selected polymers on percent drug release as well as phase behavior of cocrystal and drug during dissolution. As shown in Figure 2(a), only 30% of CBZ release occurred from matrix tablets of HPMC at 1 : 1 cocrystal to polymer ratio, while 35% and 53% of drug were released at 1 : 0.5 and 1 : 0.25 cocrystal to polymer ratios. The inverse relationship between drug release and polymer concentration is due to strong retardant effect of HPMC on release of CBZ in matrix tablets. At the end of the study, swelled intact tablets were still present in dissolution medium after six hours; samples were carefully collected, dried for 24 hours at room temperature, and crushed in mortar and pestle in order to study the phase behavior of cocrystal in matrix tablets. As stated earlier, a study has shown that HPMC prevents the conversion of CBZ III to the stable dihydrate in solution (CBZ-DH) in the gel film of tablets and in aqueous environment. Similar results were found here. In addition, HPMC inhibited the phase conversion of both drug and cocrystal in formulation at all concentration levels studied. Typical cocrystal diffraction peaks can be seen in Figure 3(a) at 5.8, 9.7, 11.5, 14.7, 22.8, and 29.9. Similarly, CBZ III characteristic peaks at 15.4 and 27.5 can also be observed in Figure 3(e) indicating stability of form 3 in matrix tablets. This inhibition effect of HPMC on crystallization is due to hydrogen bond formation between CBZ and HPMC. Moreover, the hydroxyl groups of HPMC connect to water binding sites of CBZ, thus resulting in stability of CBZ/cocrystal in dissolution medium (as shown in Figure 3(f) at 1033 cm^−1^). When similar polymer concentrations were applied to pure CBZ for comparison, drug release was slightly lesser than that from cocrystal due to higher polymer concentration than cocrystal system (as succinic acid is missing but polymer load was similar to cocrystal for comparison of equal quantity of drug).

More than twofold increase in drug release rate from Kollidon VA/64 tablets occurred at 1 : 1 cocrystal-polymer rather than at 1 : 0.25 ratio. It illustrates that Kollidon VA/64 is a good solubilizer for CBZ and can effectively be used to improve the bioavailability of drug in tablet formulations. As the concentration of polymer was decreased in formulations CSK-2 and CSK-3, drug release was also decreased considerably. At the end of experiment, tablet residue was collected for further studies. The XRD and Raman spectra confirmed that, like HPMC, Kollidon VA/64 was also capable of keeping the integrity of cocrystal in dissolution medium at all polymer concentration levels (Figure 3(b)). This inhibition effect of Kollidon VA/64 on phase transformation of CBZ has not been reported in literature, where it has mainly been used as solubilizer. At similar polymer load, drug release from pure drug (CK-1) was slightly higher than that from cocrystal (90% drug release in comparison to 83% from CSK-1).

In cocrystal-Soluplus formulations, difference in drug release at 1 : 0.5 and 1 : 0.25 ratios was nonsignificantly different, while at 1 : 1 ratio in formulation CSS-1, total 83% drug release occurred. Earlier studies have also demonstrated a direct relationship between CBZ loading and percent drug release in Soluplus solid dispersions [35]. In formulations CSS-2/CSS-3, no tablet residue was left in between 3 to 4 hours, and there was no drug release from 4th hour to 6th hour of study. Similarly, after 5th hour, tablets of formulation CSS-1 were also dissolved completely, so no residue was available for studying the phase purity of solid powder. Similar results were also observed for pure drug. The higher drug release from Kollidon VA/64 and Soluplus formulations is due to the fact that polymers having amide linkages in their molecules (N-C=O) form complexes with APIs more easily than those polymers bearing alcohol moieties (C-OH), for example, HPMC. The inhibition of crystallization is achieved through a combination of hydrophobic interactions and steric hindrance, supplemented by the ionization state and amphiphilic nature of the polymer [44].

As shown in Figure 3(f), these polymers form hydrogen bond with CBZ. Polymers with innate binding sites permit the hydrogen-bonding/van der Waals' interactions with APIs, thus leading to drug stability in the matrix system [45]. These polymers form water soluble complexes with many active substances and increase their oral bioavailability. The oral bioavailability of gidazepam was increased by the addition of Kollidon VA 64. Soluplus, a polymeric solubilizer, is a graft copolymer comprised of polyethylene glycol, polyvinyl caprolactam, and polyvinyl acetate. Soluble grades of PVP and PVP-VA copolymer have been used to improve the bioavailability of many poorly water soluble drugs like indomethacin, tolbutamide, and nifedipine [46]. This unique structure provides ideal interactions with APIs through hydrogen bonding, influences stability, and enhances solubility of drugs. However, the variability in dissolution rate is influenced by the nature of polymers, API-polymer interactions, and moisture uptake by API-polymer blends. These results indicate that drug dissolution rate from all HPMC/Kollidon VA64 formulations was more sluggish than the rate of formation of CBZ-polymer complex in solution at hydrogen-bonding interaction site, where water molecules usually attach to CBZ molecules. These results are different from recently published work of Li et al., where they reported that HPMC was incapable of stabilizing the cocrystal of CBZ-NIC even at high concentration of 1 : 1 drug to polymer ratio [47].

The dissolution profile of formulations CH followed zero-order drug release at all concentrations, while dissolution data of formulations CK followed first-order drug release kinetics. Soluplus released the drug via first-order release kinetics in CS-1 and CS-2, while in case of CS-3 it allowed the release of drug via Higuchian pattern (Table 5). In case of cocrystal-based matrices, formulations containing HPMC showed zero-order release of drug (CSH-1 and CSH-2) except for CSH-3 whereby the drug release data best fitted to first-order kinetics. Moreover, cocrystal-based matrices of Kollidon VA/64 followed first-order drug release. For cocrystal-based formulations containing Soluplus, CSS-1 followed zero order and CSS-2 followed Higuchi model, while drug release data of CSS-3 formulation best fitted to first-order release kinetics. In order to get best fit model, 60% of the drug release data was put in Korsmeyer-Peppas model [48]. Moreover, CS-2 and CS-3 formulations exhibited Fickian release (*n* < 0.45), whereas both CBZ and CBZ-SUC based formulations containing HPMCAS (except for CSH-3) followed super case-II type drug release (*n* ≥ 0.89). All the other formulations showed non-Fickian (anomalous) drug release (0.45 ≤ *n* ≤ 0.89).

## 4. Conclusion

The CBZ-SUC cocrystal shows a high aqueous solubility than its stable dihydrate form in water. Thus, use of this cocrystal system without phase transition in suitable preclinical formulation is of immense interest for bioavailability improvement of CBZ. The influence of these three commonly used polymers on the phase conversion and drug release rates of CBZ-SUC cocrystal in matrix tablets has been explored using XRPD and Raman spectroscopy. The outcomes of this study pointed out that HPMC/Kollidon VA/64 can effectively restrain the phase transition of CBZ-SUC cocrystal to CBZDH in dissolution medium or in the gel coat of the matrix tablets. In conclusion, it is certain that cocrystals can present immense benefit to fine tune physicochemical properties of existing drug molecules, yielding better solubility and dissolution of otherwise weakly water soluble APIs. But cocrystals sometimes undergo quick transitions during dissolution, which undo the pharmaceutical benefits presented by the cocrystal; it demands their robust stabilization in formulation during preformulation studies in turn for their successful use in tablet dosage form. Based on these results, combination of crystallization inhibitor (HPMC) with suitable solubilizers/surfactant (Soluplus) in a tablet formulation can dramatically improve the dissolution as well as bioavailability of CBZ.

## Figures and Tables

**Table 1:** Composition of different CBZ-SUC tablets formulations.

Composition of matrix tablet	Code	Drug : polymer	Percent drug released after six hours
CBZ-SUC: HPMC	CSH-1	1 : 1	30 ± 2.5
	CSH-2	1 : 0.5	39 ± 1.2
	CSH-3	1 : 0.25	53 ± 2.7

CBZ-SUC: Soluplus	CSS-1	1 : 1	77 ± 3.5
	CSS-2	1 : 0.5	67 ± 2.6
	CSS-3	1 : 0.25	59 ± 5.1

CBZ-SUC: Kollidon VA 64	CSK-1	1 : 1	83 ± 3.2
	CSK-2	1 : 0.5	50 ± 4.1
	CSK-3	1 : 0.25	38 ± 2.4

^*∗*^250 mg of CBZ-SUC is equivalent to 200 mg pure CBZ.

**Table 2:** Composition of different CBZ tablets formulations.

Composition of matrix tablet	Code	Drug : polymer	Percent drug released after six hours
CBZ: HPMC	CH-1	1 : 1.25	25 ± 2.3
	CH-2	1 : 0.625	36 ± 1.9
	CH-3	1 : 0.31	45 ± 2.5

CBZ: Soluplus	CS-1	1 : 1.25	83 ± 0.9
	CS-2	1 : 0.625	72 ± 1.9
	CS-3	1 : 0.31	67 ± 3.1

CBZ: Kollidon VA 64	CK-1	1 : 1.25	90 ± 2.6
	CK-2	1 : 0.625	59 ± 4.7
	CK-3	1 : 0.31	43 ± 4.1

**Table 3:** Physical evaluation of cocrystal tablets.

Formulation	Weight (mg)	Thickness (mm)	Hardness (N)	Diameter (mm)
CSH-1	497.31 ± 3.50	4.66 ± 0.01	210.34 ± 6.30	10.00 ± 0.01
CSH-2	372.04 ± 1.52	4.20 ± 0.01	200.71 ± 5.12	10.01 ± 0.01
CSH-3	309.67 ± 24	3.85 ± 0.01	197.56 ± 6.35	10.02 ± 0.01
CSS-1	495.92 ± 4.50	4.56 ± 0.01	217.34 ± 5.18	10.01 ± 0.01
CSS-2	374.39 ± 2.82	4.13 ± 0.01	205.67 ± 4.90	10.01 ± 0.00
CSS-3	311.71 ± 1.39	3.77 ± 0.01	210.07 ± 7.37	10.02 ± 0.00
CSK-1	499.19 ± 2.17	4.71 ± 0.01	197.01 ± 6.05	10.01 ± 0.00
CSK-2	308.54 ± 3.13	4.18 ± 0.01	213.21 ± 3.57	10.00 ± 0.00
CSH-3	307.76 ± 4.00	3.86 ± 0.01	208.78 ± 5.62	10.02 ± 0.01

**Table 4:** Physical evaluation of CBZ tablets.

Formulation	Weight (mg)	Thickness (mm)	Hardness (N)	Diameter (mm)
CH-1	444.64 ± 2.51	4.71 ± 0.02	198.47 ± 3.50	10.01 ± 0.00
CH-2	320.24 ± 2.60	4.28 ± 0.01	195.38 ± 6.60	10.00 ± 0.01
CH-3	258.18 ± 1.53	4.01 ± 0.03	192.72 ± 4.91	10.02 ± 0.01
CS-1	440.49 ± 2.13	4.64 ± 0.01	207.95 ± 4.36	10.02 ± 0.01
CS-2	322.25 ± 1.61	4.20 ± 0.01	209.45 ± 2.90	10.00 ± 0.01
CS-3	262.91 ± 0.01	3.84 ± 0.01	201.09 ± 3.53	9.99 ± 0.15
CK-1	449.02 ± 0.50	4.76 ± 0.03	194.46 ± 4.67	10.02 ± 0.01
CK-2	317.09 ± 3.16	4.22 ± 0.01	199.24 ± 6.10	10.01 ± 0.01
CH-3	260.11 ± 1.00	3.89 ± 0.01	203.16 ± 4.32	10.03 ± 0.01

**Table 5:** Drug release kinetics from various formulations of CBZ and CBZ-SUC.

Code	Zero order (*R* ^2^)	First order (*R* ^2^)	Higuchi model (*R* ^2^)	Korsmeyer-Peppas model		*T*_90%_ (h)
				(*R* ^2^)	(*n*)	
CH-1	0.9673	0.9603	0.8873	0.9823	1.431	24.29
CK-1	0.9395	0.9970	0.9912	0.9998	0.625	4.84
CS-1	0.7878	0.9624	0.9252	1.0000	0.812	4.93
CH-2	0.9983	0.9960	0.9509	0.9983	1.042	15.42
CK-2	0.9893	0.9991	0.9835	0.9984	0.744	8.27
CS-2	0.8204	0.9386	0.9483	0.9856	0.322	5.68
CH-3	0.9991	0.9983	0.9632	0.9996	0.932	11.78
CK-3	0.9910	0.9949	0.9786	0.9966	0.773	12.12
CS-3	0.6579	0.8227	0.8470	1.0000	0.045	5.80
CSH-1	0.9784	0.9712	0.9079	0.9869	1.290	20.53
CSK-1	0.9926	0.9927	0.9781	0.9974	0.866	6.05
CSS-1	0.9903	0.9891	0.9728	0.9987	0.768	6.39
CSH-2	0.9987	0.9977	0.9614	0.9990	0.942	13.93
CSK-2	0.9908	0.9963	0.9765	0.9964	0.794	10.43
CSS-2	0.9101	0.9781	0.9824	0.9988	0.644	6.22
CSH-3	0.9956	0.9961	0.9712	0.9977	0.846	9.95
CSK-3	0.9861	0.9949	0.9784	0.9949	0.761	12.56
CSS-3	0.9526	0.9865	0.9818	0.9843	0.597	7.41

## References

[1] Amidon G. L., Lennernas H., Shah V. P., Crison J. R. (1995). A theoretical basis for a biopharmaceutic drug classification: the correlation of in vitro drug product dissolution and in vivo bioavailability. *Pharmaceutical Research*.

[2] Panakanti R., Narang A. S. (2012). Impact of excipient interactions on drug bioavailability from solid dosage forms. *Pharmaceutical Research*.

[3] Lipinski C., Lombardo F., Dominy B., Feeney P. (1997). Experimental and computational approaches to estimate solubility and permeability in drug discovery and development settings. *Advanced Drug Delivery Reviews*.

[4] Noyes A. A., Whitney W. R. (1897). The rate of solution of solid substances in their own solutions. *The Journal of the American Chemical Society*.

[5] Blagden N., De Matas M., Gavan P. T., York P. (2007). Crystal engineering of active pharmaceutical ingredients to improve solubility and dissolution rates. *Advanced Drug Delivery Reviews*.

[6] Dai W.-G., Pollock-Dove C., Dong L. C., Li S. (2008). Advanced screening assays to rapidly identify solubility-enhancing formulations: high-throughput, miniaturization and automation. *Advanced Drug Delivery Reviews*.

[7] Serajuddin A. T. M. (2007). Salt formation to improve drug solubility. *Advanced Drug Delivery Reviews*.

[8] Serajuddln A. T. M. (1999). Solid dispersion of poorly water-soluble drugs: early promises, subsequent problems, and recent breakthroughs. *Journal of Pharmaceutical Sciences*.

[9] Bethune S. J., Huang N., Jayasankar A., Rodríguez-Hornedo N. (2009). Understanding and predicting the effect of cocrystal components and pH on cocrystal solubility. *Crystal Growth & Design*.

[10] Thakuria R., Delori A., Jones W., Lipert M. P., Roy L., Rodríguez-Hornedo N. (2013). Pharmaceutical cocrystals and poorly soluble drugs. *International Journal of Pharmaceutics*.

[11] Alhalaweh A., George S., Basavoju S., Childs S. L., Rizvi S. A. A., Velaga S. P. (2012). Pharmaceutical cocrystals of nitrofurantoin: screening, characterization and crystal structure analysis. *CrystEngComm*.

[12] Trask A. V., Motherwell W. D. S., Jones W. (2006). Physical stability enhancement of theophylline via cocrystallization. *International Journal of Pharmaceutics*.

[13] Lou B., Perumalla S. R., Sun C. C. (2015). Significant expansion of the solid state landscape of salicylic acid based on charge-assisted hydrogen bonding interactions. *Crystal Growth & Design*.

[14] Perumalla S. R., Sun C. C. (2012). Design and synthesis of solid state structures with conjugate acid-base pair interactions. *CrystEngComm*.

[15] Perumalla S. R., Sun C. C. (2014). Design and preparation of a 4:1 lamivudine-oxalic acid CAB cocrystal for improving the lamivudine purification process. *Crystal Growth & Design*.

[16] Gao Y., Gao J., Liu Z. (2012). Coformer selection based on degradation pathway of drugs: a case study of adefovir dipivoxil-saccharin and adefovir dipivoxil-nicotinamide cocrystals. *International Journal of Pharmaceutics*.

[17] Perumalla S. R., Sun C. C. (2013). Improved solid-state stability of salts by cocrystallization between conjugate acid-base pairs. *CrystEngComm*.

[18] McNamara D. P., Childs S. L., Giordano J. (2006). Use of a glutaric acid cocrystal to improve oral bioavailability of a low solubility API. *Pharmaceutical Research*.

[19] Chow S. F., Chen M., Shi L., Chow A. H. L., Sun C. C. (2012). Simultaneously improving the mechanical properties, dissolution performance, and hygroscopicity of ibuprofen and flurbiprofen by cocrystallization with nicotinamide. *Pharmaceutical Research*.

[20] Krishna G. R., Shi L., Bag P. P., Sun C. C., Reddy C. M. (2015). Correlation among crystal structure, mechanical behavior, and tabletability in the co-crystals of vanillin isomers. *Crystal Growth & Design*.

[21] Levy R. H., Pitlick W. H., Troupin A. S., Green J. R., Neal J. M. (1975). Pharmacokinetics of carbamazepine in normal man. *Clinical Pharmacology and Therapeutics*.

[22] Moneghini M., Voinovich D., Perissutti B., Princivalle F. (2002). Action of carriers on carbamazepine dissolution. *Pharmaceutical Development and Technology*.

[23] Murphy D., Rodríguez-Cintrón F., Langevin B., Kelly R. C., Rodríguez-Hornedo N. (2002). Solution-mediated phase transformation of anhydrous to dihydrate carbamazepine and the effect of lattice disorder. *International Journal of Pharmaceutics*.

[24] Tian F., Zeitler J. A., Strachan C. J., Saville D. J., Gordon K. C., Rades T. (2006). Characterizing the conversion kinetics of carbamazepine polymorphs to the dihydrate in aqueous suspension using Raman spectroscopy. *Journal of Pharmaceutical and Biomedical Analysis*.

[25] Chieng N., Hubert M., Saville D., Rades T., Aaltonen J. (2009). Formation kinetics and stability of carbamazepine-nicotinamide cocrystals prepared by mechanical activation. *Crystal Growth and Design*.

[26] Childs S. L., Rodríguez-Hornedo N., Reddy L. S. (2008). Screening strategies based on solubility and solution composition generate pharmaceutically acceptable cocrystals of carbamazepine. *CrystEngComm*.

[27] Hickey M. B., Peterson M. L., Scoppettuolo L. A. (2007). Performance comparison of a co-crystal of carbamazepine with marketed product. *European Journal of Pharmaceutics and Biopharmaceutics*.

[28] Rodríguez-Hornedo N., Murphy D. (2004). Surfactant-facilitated crystallization of dihydrate carbamazepine during dissolution of anhydrous polymorph. *Journal of Pharmaceutical Sciences*.

[29] Li M., Qiao N., Wang K. (2013). Influence of sodium lauryl sulfate and Tween 80 on carbamazepine-nicotinamide cocrystal solubility and dissolution behaviour. *Pharmaceutics*.

[30] Qiao N., Wang K., Schlindwein W., Davies A., Li M. (2013). In situ monitoring of carbamazepine–nicotinamide cocrystal intrinsic dissolution behaviour. *European Journal of Pharmaceutics and Biopharmaceutics*.

[31] Raghavan S. L., Schuessel K., Davis A., Hadgraft J. (2003). Formation and stabilisation of triclosan colloidal suspensions using supersaturated systems. *International Journal of Pharmaceutics*.

[32] Simonelli A. P., Mehta S. C., Higuchi W. I. (1970). Inhibition of sulfathiazole crystal growth by polyvinylpyrrolidone. *Journal of Pharmaceutical Sciences*.

[33] Terayama H., Inada K., Nakayama H., Yasueda S., Esumi K. (2004). Preparation of stable aqueous suspension of a hydrophobic drug with polymers. *Colloids and Surfaces B: Biointerfaces*.

[34] Katzhendler I., Azoury R., Friedman M. (1998). Crystalline properties of carbamazepine in sustained release hydrophilic matrix tablets based on hydroxypropyl methylcellulose. *Journal of Controlled Release*.

[35] Hardung H., Djuric D., Ali S. (2010). Combining HME & solubilization: Soluplus—the solid solution. *Drug Development & Delivery*.

[36] Childs S. L., Kandi P., Lingireddy S. R. (2013). Formulation of a danazol cocrystal with controlled supersaturation plays an essential role in improving bioavailability. *Molecular Pharmaceutics*.

[37] Friesen D. T., Shanker R., Crew M., Smithey D. T., Curatolo W. J., Nightingale J. A. S. (2008). Hydroxypropyl methylcellulose acetate succinate-based spray-dried dispersions: an overview. *Molecular Pharmaceutics*.

[38] Tomaszewska I., Karki S., Shur J., Price R., Fotaki N. (2013). Pharmaceutical characterisation and evaluation of cocrystals: importance of in vitro dissolution conditions and type of coformer. *International Journal of Pharmaceutics*.

[39] Costa P., Lobo J. M. S. (2001). Modeling and comparison of dissolution profiles. *European Journal of Pharmaceutical Sciences*.

[40] Gibaldi M., Feldman S. (1967). Establishment of sink conditions in dissolution rate determinations. Theoretical considerations and application to nondisintegrating dosage forms. *Journal of Pharmaceutical Sciences*.

[41] Wagner J. G. (1969). Interpretation of percent dissolved-time plots derived from in vitro testing of conventional tablets and capsules. *Journal of Pharmaceutical Sciences*.

[42] Higuchi T. (1963). Mechanism of sustained-action medication. Theoretical analysis of rate of release of solid drugs dispersed in solid matrices. *Journal of Pharmaceutical Sciences*.

[43] Peppas N. A. (1985). Analysis of Fickian and non-Fickian drug release from polymers. *Pharmaceutica Acta Helvetiae*.

[44] Dinunzio J. C., Hughey J. R., Brough C., Miller D. A., Williams R. O., McGinity J. W. (2010). Production of advanced solid dispersions for enhanced bioavailability of itraconazole using KinetiSol Dispersing. *Drug Development and Industrial Pharmacy*.

[45] Alonzo D. E., Zhang G. G. Z., Zhou D., Gao Y., Taylor L. S. (2010). Understanding the behavior of amorphous pharmaceutical systems during dissolution. *Pharmaceutical Research*.

[46] Qian F., Huang J., Hussain M. A. (2010). Drug-polymer solubility and miscibility: stability consideration and practical challenges in amorphous solid dispersion development. *Journal of Pharmaceutical Sciences*.

[47] Li M., Qiu S., Lu Y., Wang K., Lai X., Rehan M. (2014). Investigation of the effect of hydroxypropyl methylcellulose on the phase transformation and release profiles of carbamazepine-nicotinamide cocrystal. *Pharmaceutical Research*.

[48] Murtaza G., Ullah H., Khan S. A. (2015). Formulation and in vitro dissolution characteristics of sustained-release matrix tablets of tizanidine hydrochloride. *Tropical Journal of Pharmaceutical Research*.

